# Supplementary material for: Genomic landscape and clonal architecture of mouse oral squamous cell carcinomas dictate tumour ecology
Source: Nat Commun. 2020 Nov 9;11:5671. doi: 10.1038/s41467-020-19401-9 (PMC7652942; doi:10.1038/s41467-020-19401-9)
Supplement: Supplementary file 1 — Supplementary Information [file 41467_2020_19401_MOESM1_ESM.pdf]

Supplementary Information

GENOMIC LANDSCAPE AND CLONAL ARCHITECTURE OF MOUSE ORAL SQUAMOUS CELL CARCINOMAS DICTATE TUMOUR ECOLOGY

Sequeira *et al.*

Supplementary Figures 1- 5

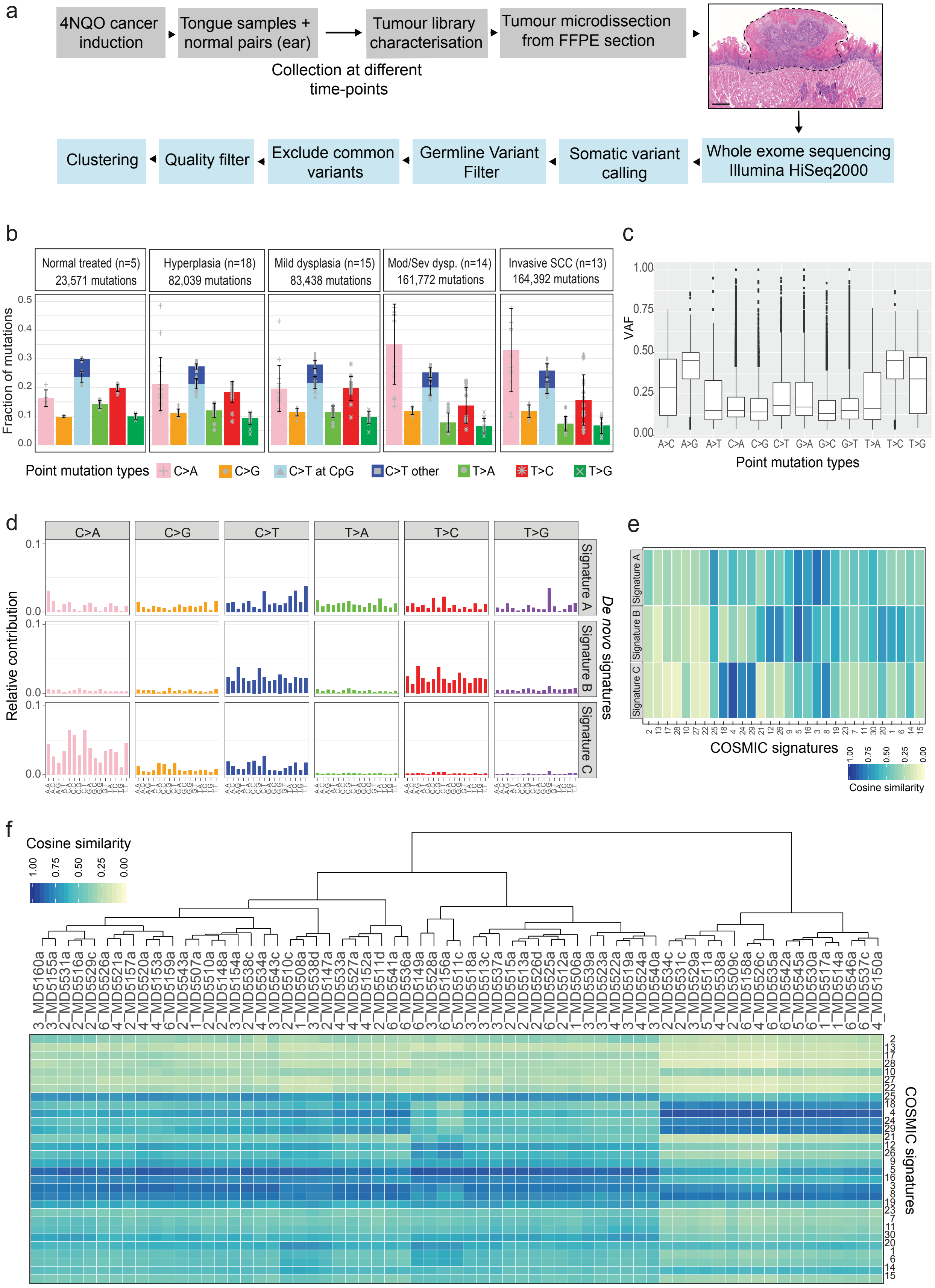

Supplementary Figure 1. Detailed experimental design and tumour grading and Mutational signature analysis. Related to Figure 1. (a) Experimental design and analytical workflow for calling somatic variants. Sample preparation (grey): the tumours identified were preserved in FFPE blocks. All

animals had a normal matched control tissue harvested from the ear. Tumour histology features were observed microscopically and characterised. Tumour region was micro-dissected avoiding healthy tissue contamination. Both FFPE blocks and matched control tissue were sent for WES. Genomic Analysis (light blue): sequencing data was filtered for inter-individual variability with somatic calling. Filtering, clustering and data validation was completed. **(b)** Mutational signatures for each tumour grading, from normal tissue treated, hyperplasia, mild dysplasia, moderate/severe dysplasia and invasive SCCs. Error bars indicate standard deviation over all samples. **(c)** Frequencies for each of the nucleotide substitutions are displayed as boxplots (the middle line is the median, the lower and upper hinges are the first and third quartiles, the upper whisker extends from the hinge to the largest value no further than 1.5× inter-quartile range from the hinge and the lower whisker extends from the hinge to the smallest value at most 1.5× inter-quartile range of the hinge. Data beyond the end of the whiskers are outliers that are plotted individually). **(d)** 96-profile of the three *de novo* signatures. **(e)** Similarity heatmap featuring the contribution of each *De novo* signature to the known COSMIC signatures[32]. **(f)** Similarity heatmap featuring the contribution of known COSMIC signatures for all the samples.

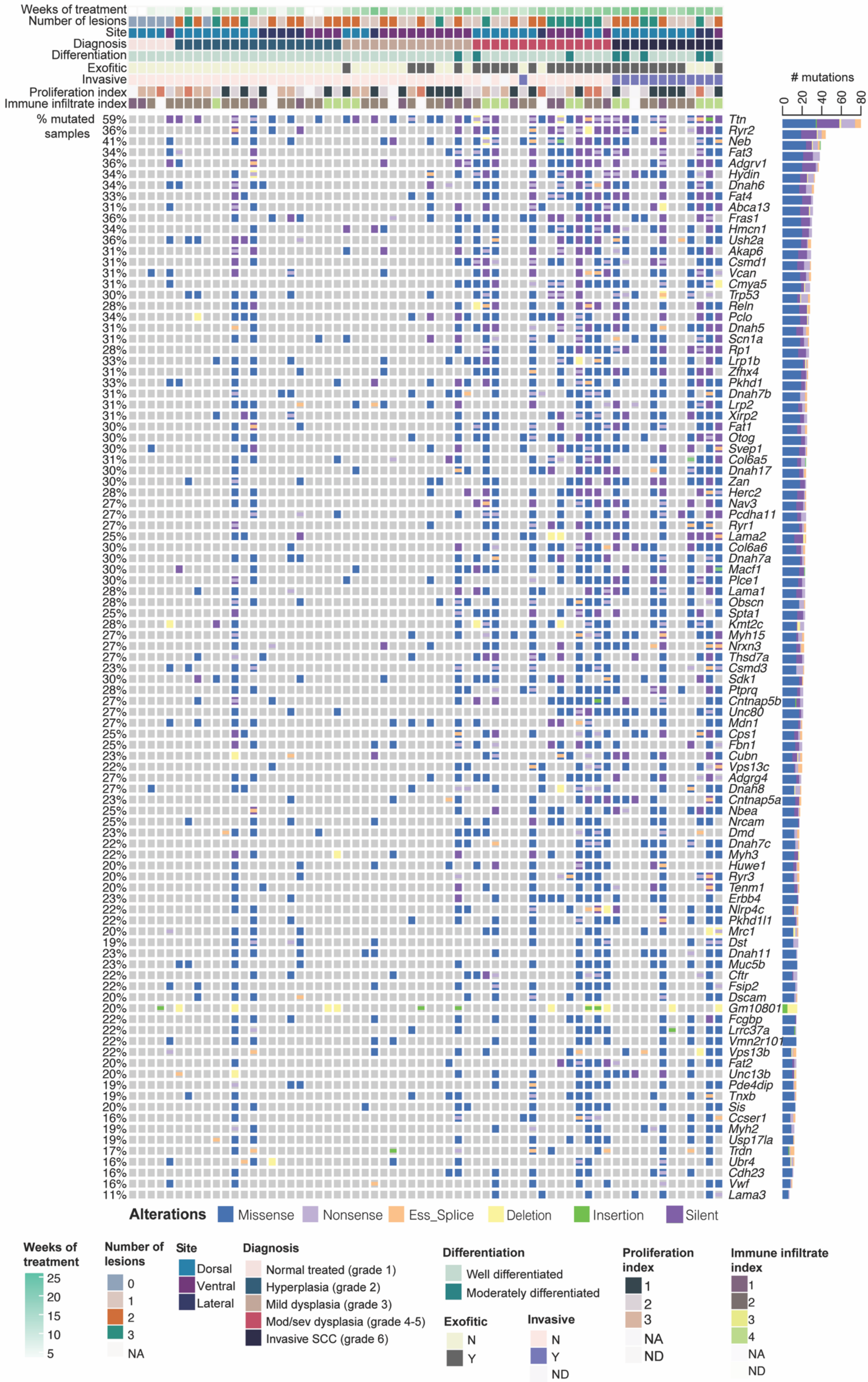

Weeks of treatment
Number of lesions
Site
Diagnosis
Differentiation
Exofitic
Invasive
Proliferation index
Immune infiltrate index
% mutated samples
# mutations
Ttn
Ryr2
Neb
Fat3
Adgrv1
Hydin
Dnah6
Fat4
Abca13
Fras1
Hmcn1
Ush2a
Akap6
Csmd1
Vcan
Cmya5
Trp53
Reln
Pclo
Dnah5
Scn1a
Rp1
Lrp1b
Zfhx4
Pkhd1
Dnah7b
Lrp2
Xirp2
Fat1
Otog
Svep1
Col6a5
Dnah17
Zan
Herc2
Nav3
Pcdha11
Ryr1
Lama2
Col6a6
Dnah7a
Macf1
Plce1
Lama1
Obscn
Spta1
Kmt2c
Myh15
Nrxn3
Thsd7a
Csmd3
Sdk1
Ptprq
Cntnap5b
Unc80
Mdn1
Cps1
Fbn1
Cubn
Vps13c
Adgrg4
Dnah8
Cntnap5a
Nbea
Nrcam
Dmd
Dnah7c
Myh3
Huwe1
Ryr3
Tenm1
Erbb4
Nlrp4c
Pkhd1l1
Mrc1
Dst
Dnah11
Muc5b
Cftr
Fsip2
Dscam
Gm10801
Fcgbp
Lrrc37a
Vmn2r101
Vps13b
Fat2
Unc13b
Pde4dip
Tnxb
Sis
Ccser1
Myh2
Usp17la
Trdn
Ubr4
Cdh23
Vwf
Lama3
Alterations
Missense
Nonsense
Ess_Splice
Deletion
Insertion
Silent
Weeks of treatment
Number of lesions
Site
Dorsal
Ventral
Lateral
Diagnosis
Normal treated (grade 1)
Hyperplasia (grade 2)
Mild dysplasia (grade 3)
Mod/sev dysplasia (grade 4-5)
Invasive SCC (grade 6)
Differentiation
Well differentiated
Moderately differentiated
Exofitic
Invasive
Proliferation index
Immune infiltrate index

Supplementary Figure 2. Data matrix showing mutational landscape of 4NQO-induced TSCCs including recurrent somatic mutations compared with tumour ecology. Related to Figure 2. The top panel shows the key clinical parameters: weeks of 4NQO treatment, number of lesions per tongue, tumour-site (dorsal, ventral, lateral), diagnosis (tumour-grade), differentiation status, exophytic tumour (yes/no), invasive tumour (yes/no), proliferation index, immune-infiltrate index and type of SNV – 'alterations' (Supplementary Data 1, 2 and 3). The bottom panel shows the plot of significantly mutated genes from our samples. Representation of the mutations (n=1177) in the top 100 most mutated genes coloured by the types of mutation. Each column denotes an individual sample (n=65), and each row represents a gene. Genes are ordered by level of significance. The right panel shows the number of mutations in each gene. Percentages represent the fraction of tumours harbouring mutations in the corresponding gene.

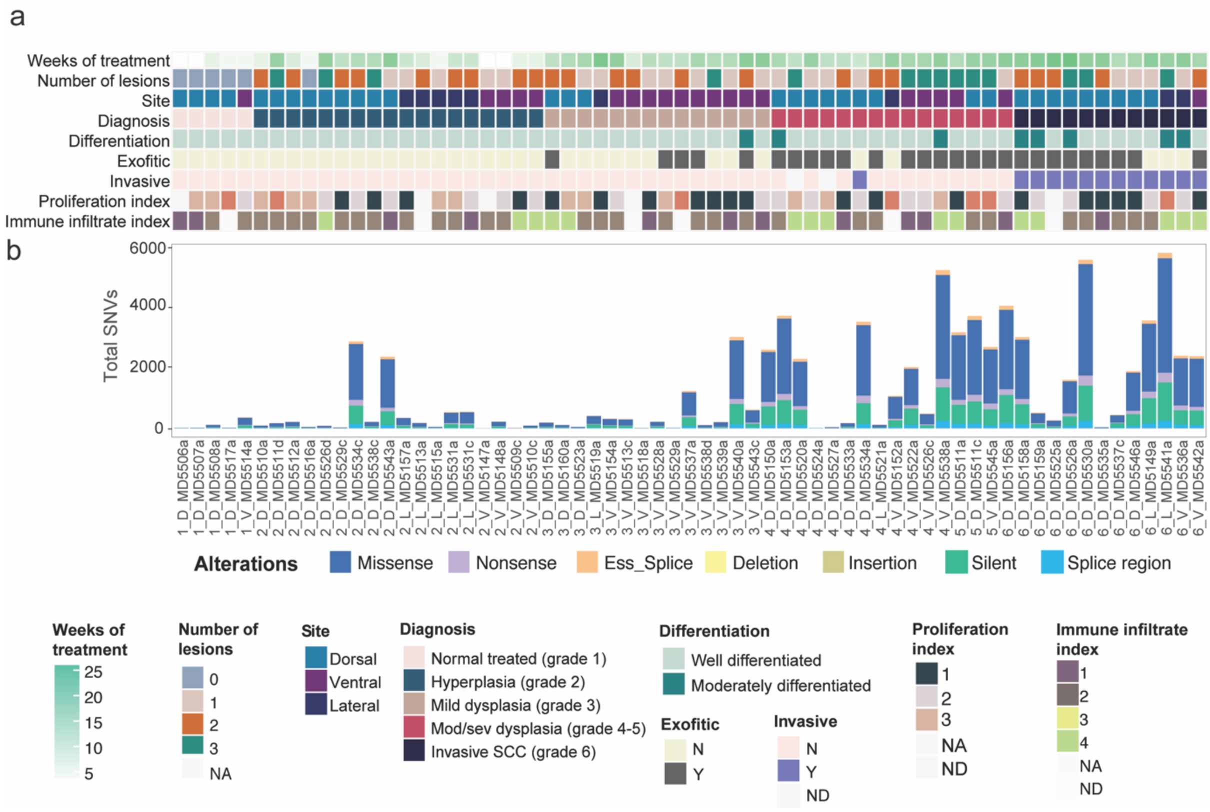

Supplementary Figure 3. **Correlation between mutation burden and TSCCs tumour ecology. (a)** Key clinical parameters (as in Figure 2): colour coding indicates number of weeks of treatment, number of lesions per tongue, site of the sequenced lesion (dorsal, ventral or lateral), diagnosis or tumour-grade, differentiation status, exophytic tumour (yes/no), invasive tumour (yes/no), proliferation index, immune-infiltrate index (CD45) and type of SNV (Supplementary Tables 1, 2 and 3). **(b)** Barplot showing the number of mutations per sample. All missense and nonsense SNVs, splicing mutations (Exonic Splicing Silencer 'Ess_Splice' and splice regions), insertions, deletions and silent mutations in genes listed in Supplementary Data 3 and Figure 2 are displayed.

a b c

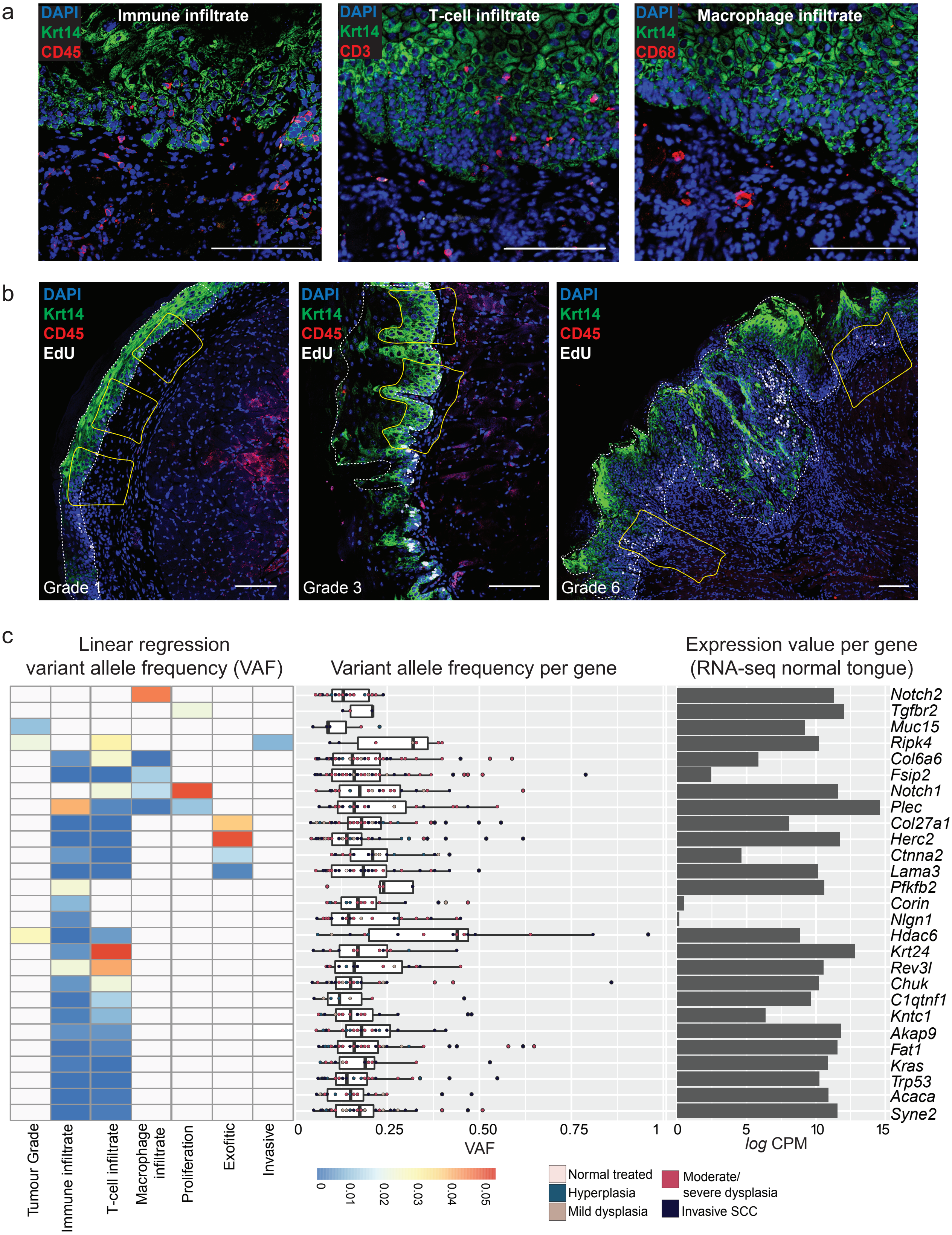

d

Total mutations

	Predictors	Estimates	CI	p-value
**All genes**	Exofitic	1302.997	08.92 – 1897.06	<0.001
	Proliferation	0.99	-1.78 – -0.21	0.017
	Tumour grade	404.25	220.75 – 587.76	<0.001

Supplementary Figure 4. **Correlation between mutational landscape and tumour ecology.** Related to Figure 5. **(a)** Immunofluorescence representative images of immune infiltrate labelling against $CD45^+$ total immune cells, $CD3^+$ T-cell infiltrate or $CD68^+$ macrophage infiltrate (red), anti-Krt14 (green) and counterstained with nuclear dye DAPI (blue). **(b)** Immunofluorescence representative images illustrating the quantification of immune infiltrate and proliferation. Each tumour was labelled with anti-Krt14 (green), anti-CD45 (red), anti-EdU (white) and counterstained with nuclear dye DAPI (blue). Dividing cells (EdU^+ cells) in the epithelium were quantified within the $Krt14^+$ region and density calculated by number of EdU^+ cells/mm^2 (white doted regions). Immune infiltrate was quantified by the number of $CD45^+$ (or $CD3^+$ or $CD68^+$) cells/mm^2 in tumour stromal region and epithelial basal layers (yellow lined regions). All tumours were analysed and for each tumour ≥6 regions (white doted regions) were quantified per section (n=2-3 sections/tumour/staining). $CD45^+$ immune infiltrate index corresponds to <2,000 (index 1), 2,000-5,000 (index 2), 5,000-10,000 (index 3), >10,000 cells/mm^2 (index 4) (Supplementary Table 1). **(c)** Linear regression analysis to establish the relationship between the mutation VAF value (heatmap) of each gene (rows) and the clinical parameters (columns) (Supplementary Table 7). The boxplots on the middle depict the average VAF values for each gene (the middle line is the median, the lower and upper hinges are the first and third quartiles, the upper whisker extends from the hinge to the largest value no further than 1.5 × inter-quartile range from the hinge and the lower whisker extends from the hinge to the smallest value at most 1.5 × inter-quartile range of the hinge. Data beyond the end of the whiskers are outliers that are plotted individually). Each dot represents a mutation colour-coded according to the diagnosis of the sample where the mutation occurs. The histogram on the right represents the gene expression values from RNA-sequencing data. **(d)** Linear regression analysis of total number of mutations according to clinical parameters (tumour grade, tumour proliferation and exofitic tumour) for all genes. Predictors, Estimates, Confidence Interval (CI) and *p* values. Scale bars: 100μm (a), 200μm (b).

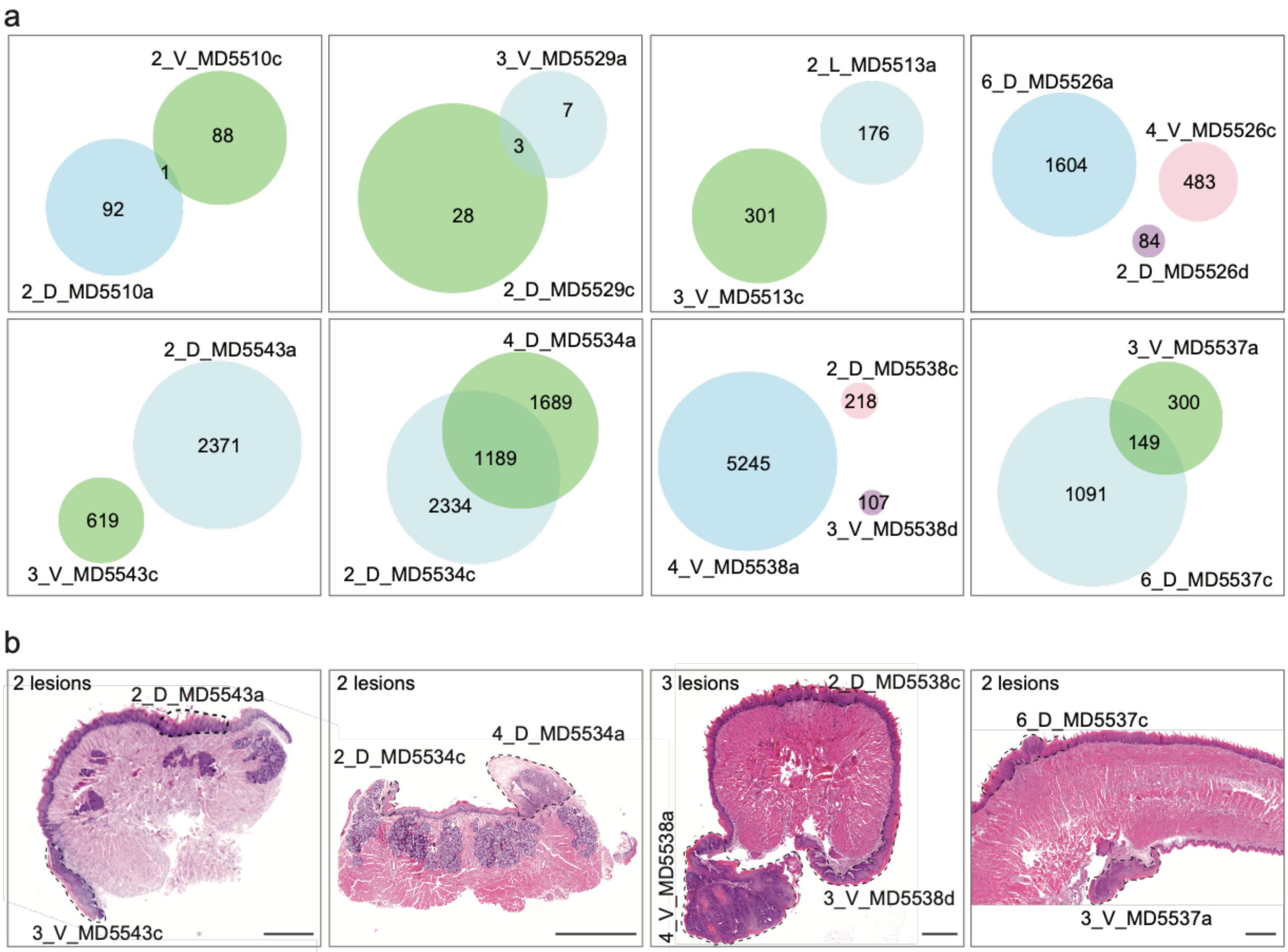

Supplementary Figure 5. **Comparative analysis of synchronous lesions in the same animal. (a)** VENN diagrams comparing the total numbers of mutations between two or three lesions from the same animals. **(b)** Representative sequential tumour sections sequential from the microdissected FFPE sections used for sequencing and stained with haematoxylin-eosin. Doted lines represent the microdissected regions sequenced. n=6 animals with two synchronous lesions sequenced; n=2 animals with three synchronous lesions sequenced. Each number corresponds to the tumour reference: 2_D_MD5534c; 2, 3, 4 or 6=tumour grade, D=dorsal, V=ventral.

Supplementary Table 1 – Clinical and biological data analysed in each tongue sample.

Parameters analysed	Classification	Method description
Weeks of treatment	4 – 26	Number of weeks after starting treatment administration (Figure 1a). Short (1-9 weeks), Medium (10-17 weeks) and Long treatment (18-26 weeks).
Lesions per animal	1 – 3	Number of lesions/tumors in each animal's tongue.
Site	D, L, V	Dorsal, lateral, ventral (Figure 1b)
Lesion grading	1, 2, 3, 4, 5, 6	Ascendant grading indicates increased abnormalities in the tissue. Numbers correspond to normal-treated tissue (1), hyperplasia (2), mild (3), moderate (4), or severe dysplasia (5) and invasive SCC (6) (Figure 1d).
Lesion dimensions	Length (μm), maximum diameter (μm), maximum invasiveness (μm)	Measurements were done using *NanoZoomer* Digital Pathology Software. Length corresponds to the tumour size along the basement membrane. Diameter was measured in 3 – 6 graded lesions and when applicable. Invasiveness length, when applicable, was measured from the basement membrane.
Tumour exophitic growth	Y (Round, papilloma, verrucous), N	Assessments were made on observation of the H&E sections (Figure 1d). Y stands for Yes, N for No.
Tumour invasiveness	Y, N	Assessments were made on observation of the H&E sections. Y stands for Yes, N for No.
Lesion differentiation	Well-differentiated, moderately differentiated	Assessments were made on observation of Krt14 immunofluorescence staining (Supplementary Figure 5)
Immune infiltrate	1 - 4	Quantification of the $CD45^+$ cells in tumour stroma. Index stands for <2 000 (1), 2 000 - 5 000 (2), 5 000 - 10 000 (3), >10 000 cells/mm^2 (4) (Figure 5 and Supplementary Figure 5).
T-cell infiltrate	1 - 3	Quantification of the $CD3^+$ cells in tumour stroma. Index stands for <500 (1), 500 – 1 000 (2), >1 000 cells/mm^2 (3) (Figure 5 and Supplementary Figure 5).
Macrophage infiltrate	1 - 3	Quantification of the $CD68^+$ cells in tumour stroma. Index stands for 0 – 50 (1), 51 – 100 (2), >100 cells/mm^2 (3) (Figure 5 and Supplementary Figure 5).
Proliferation	1 - 3	Quantification of EdU^+ cells in the $Krt14^+$ microdissected tumour region (Figure 5 and Supplementary Figure 5). Index stands for <150 (1), 150 – 600 (2), >600 (3) cells/mm^2.
