# Supplementary material for: Genomic landscape and clonal architecture of mouse oral squamous cell carcinomas dictate tumour ecology
Source: Nat Commun. 2020 Nov 9;11:5671. doi: 10.1038/s41467-020-19401-9 (PMC7652942; doi:10.1038/s41467-020-19401-9)
Supplement: Supplementary file 3 — Description of Additional Supplementary Files [file 41467_2020_19401_MOESM3_ESM.pdf]

Description of Supplementary Data Files

File Name: **Supplementary Data 1**

Legend: Clinical data for all sequenced tumours and matched controls.

File Name: **Supplementary Data 2**

Legend: Matrix with WES results including all mutated genes in mouse TSCCs. Table containing the selected mutations (Missense, Nonsense, Silent, Ess_splice, Indel, Deletions, Start Lost and Stop Lost) in each gene (row) in each sample (columns). Comma separated mutations indicate multiple events present in the same gene in the same sample.

File Name: **Supplementary Data 3**

Legend: Tongue recurrent mutated genes in human TSCC. List of 465 human genes evaluated from target-exome sequencing.

File Name: **Supplementary Data 4**

Legend: *DNdScv* analysis output: annotation of mutations per sample. The first table contains the list of driver genes identified with the algorithm. For each gene, the following informations are indicated: number of mutations (synonymous, missense, nonsense, essential splice sites), maximum-likelihood estimates (MLEs) of the dN/dS ratios for each gene for each type of mutations, *p*-values and *q*-values. The second table contains the annotation for each mutation in each sample. For each mutation, the following info is indicated: sample and gene where the mutation occurs, chromosome and position, aminoacid, nucleotide and codon change, impact.

File Name: **Supplementary Data 5**

Legend: Gene Ontology analysis of the significant genes from dndscv analysis. List of 25 GO terms (biological process) for which a statistically significant enrichment was detected in the driver genes. For each category, *p*-values, FDR and enrichment score are indicated. The enrichment score is calculated as **(b/n)/(B/N)**, where: **b** is the number of genes in the intersection; **n** is the number of genes in the top of the user's input list or in the target set when appropriate; **B** is the total number of genes associated with a specific GO term; **N** is the total number of genes. The statistical test used for enrichment analysis is typically based on a hypergeometric model.

File Name: **Supplementary Data 6**

Legend: Linear regression analysis to establish the relationship between the total number of mutations and the VAF value in each gene and the clinical parameters (rows). The estimates for the model's coefficients, the standard deviation of the sampling distribution of the estimate of the coefficient under the standard regression assumptions, the *tvalue* and two-sided *p*-value (*Pr(*>|*t*|*)*) are shown for each correlation (gene versus clinical parameter).
