# Supplementary material for: Genomic landscape and clonal architecture of mouse oral squamous cell carcinomas dictate tumour ecology
Source: Nat Commun. 2020 Nov 9;11:5671. doi: 10.1038/s41467-020-19401-9 (PMC7652942; doi:10.1038/s41467-020-19401-9)
Supplement: Supplementary file 10 — Reporting Summary [file 41467_2020_19401_MOESM10_ESM.pdf]

nature research

Corresponding author(s): Fiona M Watt

Last updated by author(s): 19/08/20

Reporting Summary

Nature Research wishes to improve the reproducibility of the work that we publish. This form provides structure for consistency and transparency in reporting. For further information on Nature Research policies, see Authors & Referees and the Editorial Policy Checklist .

Statistics

For all statistical analyses, confirm that the following items are present in the figure legend, table legend, main text, or Methods section.

n/a	Confirmed	
☐	☒	The exact sample size (*n*) for each experimental group/condition, given as a discrete number and unit of measurement
☐	☒	A statement on whether measurements were taken from distinct samples or whether the same sample was measured repeatedly
☐	☒	The statistical test(s) used AND whether they are one- or two-sided<br>*Only common tests should be described solely by name; describe more complex techniques in the Methods section.*
☒	☐	A description of all covariates tested
☒	☐	A description of any assumptions or corrections, such as tests of normality and adjustment for multiple comparisons
☐	☒	A full description of the statistical parameters including central tendency (e.g. means) or other basic estimates (e.g. regression coefficient) AND variation (e.g. standard deviation) or associated estimates of uncertainty (e.g. confidence intervals)
☐	☒	For null hypothesis testing, the test statistic (e.g. *F*, *t*, *r*) with confidence intervals, effect sizes, degrees of freedom and *P* value noted<br>*Give P values as exact values whenever suitable.*
☒	☐	For Bayesian analysis, information on the choice of priors and Markov chain Monte Carlo settings
☒	☐	For hierarchical and complex designs, identification of the appropriate level for tests and full reporting of outcomes
☒	☐	Estimates of effect sizes (e.g. Cohen's *d*, Pearson's *r*), indicating how they were calculated

Our web collection on statistics for biologists contains articles on many of the points above.

Software and code

Policy information about availability of computer code

Data collection	no software was used
Data analysis	Somatic variants were detected using CaVEMan (v1.13.14).<br>Small insertion and deletion (indel) detection was performed using the cgp-pindel pipeline (v0.2.4w)<br>All the analyses in the manuscript were performed using R bioconductor (R version 3.5.1):<br>- MutationalPatterns package (v1.8.0 and v1.10.0)<br>- dNdScv package (v0.0.1.0)<br>- ggplot2 (v3.3.0)<br>- ComplexHeatmap (v1.20.0)<br>External online tools used for enrichment analyses:<br>- GOrilla tool for Gene Ontology analysis<br>- Shiny GO for KEGG analysis<br>Custom R scripts for data manipulation and data visualization. The source data for all figures and supplementary figures are available as Source Data file. All the other data and codes used for the analysis are available from the corresponding author upon reasonable request.

For manuscripts utilizing custom algorithms or software that are central to the research but not yet described in published literature, software must be made available to editors/reviewers. We strongly encourage code deposition in a community repository (e.g. GitHub). See the Nature Research guidelines for submitting code & software for further information.

Data

Policy information about availability of data

All manuscripts must include a data availability statement. This statement should provide the following information, where applicable:

- Accession codes, unique identifiers, or web links for publicly available datasets
- A list of figures that have associated raw data
- A description of any restrictions on data availability

All TSCC whole-exome sequencing data generated have been deposited in the European Nucleotide Archive (ENA) under the study accession number: PRJEB32924. The detailed information of lesions grading and matched samples are listed in the Supplementary table 1.
Publicly available databases or resources used in this research are as follow: COSMIC (http://cancer.sanger.ac.uk/cancergenome/projects/cosmic/), Gene Ontology (http://geneontology.org/), gene expression during 4NQO treatment (GSE5426)23. All datasets used to analyse recurrent mutated genes in human TSCCs are listed in Supplementary Table 4. All other remaining data are available within the Article and Supplementary Files, or available from the authors upon request.

Field-specific reporting

Please select the one below that is the best fit for your research. If you are not sure, read the appropriate sections before making your selection.

☒ Life sciences ☐ Behavioural & social sciences ☐ Ecological, evolutionary & environmental sciences

For a reference copy of the document with all sections, see nature.com/documents/nr-reporting-summary-flat.pdf

Life sciences study design

All studies must disclose on these points even when the disclosure is negative.

Sample size	The total number of animals to treat and tumour to sequence was determine by power calculation: we estimated of the number of samples needed to be sequenced in order to identify a recurrent mutation at a given frequency with 90% power. For example, in order to detect Notch1 mutations that were described to occur in 10% of human HNSCC (cf. Cancer Genome Atlas Network. Comprehensive genomic characterization of head and neck squamous cell carcinomas. Nature 517, 576–582 (2015)), we would need to sample approximately 23 samples.
Data exclusions	No samples were excluded after sequencing data was generated. No data was excluded from this analysis.
Replication	Sequencing of lesions of animals from 6 different cohorts were analysed together. All immunostaining experiments were performed at least in 3 independent replicates and the findings were replicated. Histopathology analysis and quantifications were performed by 2 separate researchers. All attempts at experimental replication were successful.<br>All other experiments and analyses were replicated independently at least twice.
Randomization	Animals from different cages, but within the same experimental group, were selected to assure randomization.
Blinding	The authors were blinded to sample identity during data collection, data quantification and data analysis.

Reporting for specific materials, systems and methods

We require information from authors about some types of materials, experimental systems and methods used in many studies. Here, indicate whether each material, system or method listed is relevant to your study. If you are not sure if a list item applies to your research, read the appropriate section before selecting a response.

Materials & experimental systems

n/a	Involved in the study
☐	☒ Antibodies
☒	☐ Eukaryotic cell lines
☒	☐ Palaeontology
☐	☒ Animals and other organisms
☒	☐ Human research participants
☒	☐ Clinical data

Methods

n/a	Involved in the study
☒	☐ ChIP-seq
☒	☐ Flow cytometry
☒	☐ MRI-based neuroimaging

Antibodies

Antibodies used	Primary antibodies used: Rat anti-CD68 (Abcam, #ab53444, clone FA-11, Lot GR3241798-4), Rabbit anti-CD3 (Abcam, #ab16669, clone SP7, lot GR3285725-6), Rat anti-CD45 (BD Pharmingen #553076, clone 30-F11, lot 3340994), Rat anti-CD45 (Biolegend #103101, clone 30-F11, lot B165376), Rabbit anti-Krt14 (Covance #905301, PRB-155P, clone Poly19053), Chicken anti-Krt14 (Biolegend #906001, 1/5000), clone Poly9060, lot B299611), Rabbit anti-laminin a3 (gift from Matthew Caley, clone R14), Rabbit

	anti-p53 (Novocastra NCL-L-p53-CM5p, clone Poly, lot 6063635).<br>To reveal primary antibodies signal, we used conjugated secondary antibodies AlexaFluor488 goat anti-chicken (#A11039), AlexaFluor555 donkey anti-rabbit (#A31572), AlexaFluor594 goat anti-rat (#A11007), AlexaFluor647 chicken anti-rat (#A21472), diluted at a ratio of 1:300 (Invitrogen, purchased from ThermoFisher Scientific).
Validation	All Antibodies were published and validated in previous studies. All commercial antibodies were validated for the species (mouse) and applications (immunostaining) by the correspondent manufacturer, which is described in the manufacturer's website. Rabbit anti-laminin a3 was previously validated (Pesch et al., J Investig Dermatol, 2017).<br>Our usage was described in the Methods section of the manuscript.

Animals and other organisms

Policy information about studies involving animals; ARRIVE guidelines recommended for reporting animal research

Laboratory animals	WT mice with C57Bl/6N genetic background and mice aged 6~12 weeks of both genders were used for experiments. Mice were housed under 12 light/12 dark cycle, temperatures of 22±2°C with 50±10% humidity.
Wild animals	The study did not involve wild animals.
Field-collected samples	No field collected samples were used in the study.
Ethics oversight	All animal procedures were subject to institutional ethical review and were approved by the UK Home Office (in accordance with UK law, Animals Scientific Procedures Act 1986) at King's College London prior to commencement.

Note that full information on the approval of the study protocol must also be provided in the manuscript.
